# *Phragmanthera austroarabica* A.G.Mill. and J.A.Nyberg Triggers Apoptosis in MDA-MB-231 Cells In Vitro and In Vivo Assays: Simultaneous Determination of Selected Constituents

**DOI:** 10.3390/metabo12100921

**Published:** 2022-09-29

**Authors:** Marwa S. Goda, Sameh S. Elhady, Mohamed S. Nafie, Hanin A. Bogari, Raina T. Malatani, Rawan H. Hareeri, Jihan M. Badr, Marwa S. Donia

**Affiliations:** 1 Department of Pharmacognosy, Faculty of Pharmacy, Suez Canal University, Ismailia 41522, Egypt; 2 Department of Pharmacognosy, Faculty of Pharmacy, Galala University, New Galala 43713, Egypt; 3 Department of Natural Products, Faculty of Pharmacy, King Abdulaziz University, Jeddah 21589, Saudi Arabia; 4 Department of Chemistry, Faculty of Science, Suez Canal University, Ismailia 41522, Egypt; 5 Department of Pharmacy Practice, Faculty of Pharmacy, King Abdulaziz University, Jeddah 21589, Saudi Arabia; 6 Department of Pharmacology and Toxicology, Faculty of Pharmacy, King Abdulaziz University, Jeddah 21589, Saudi Arabia

**Keywords:** sustainability of natural resources, *Phragmanthera austroarabica*, HPTLC, apoptosis, MDA-MB-231 cells, drug discovery

## Abstract

*Phragmanthera austroarabica* (Loranthaceae), a semi-parasitic plant, is well known for its high content of polyphenols that are responsible for its antioxidant and anti-inflammatory activities. Gallic acid, catechin, and methyl gallate are bioactive metabolites of common occurrence in the family of Loranthaceae. Herein, the concentrations of these bioactive metabolites were assessed using high-performance thin layer chromatography (HPTLC). Methyl gallate, catechin, and gallic acid were scanned at 280 nm. Their concentrations were assessed as 14.5, 6.5 and 43.6 mg/g of plant dry extract, respectively. *Phragmanthera austroarabica* extract as well as the three pure compounds were evaluated regarding the cytotoxic activity. The plant extract exhibited promising cytotoxic activity against MDA-MB-231 breast cells with the IC_50_ value of 19.8 μg/mL while the tested pure compounds displayed IC_50_ values in the range of 21.26–29.6 μg/mL. For apoptosis investigation, *P. austroarabica* induced apoptotic cell death by 111-fold change and necrosis by 9.31-fold change. It also activated the proapoptotic genes markers and inhibited the antiapoptotic gene, validating the apoptosis mechanism. Moreover, in vivo studies revealed a significant reduction in the breast tumor volume and weight in solid Ehrlich carcinoma (SEC) mice. The treatment of SEC mice with *P. austroarabica* extract improved both hematological and biochemical parameters with amelioration in the liver and kidney histopathology to near normal. Taken together, *P. austroarabica* extract exhibited promising anti-cancer activity through an apoptosis-induction.

## 1. Introduction

**Figure 1:**
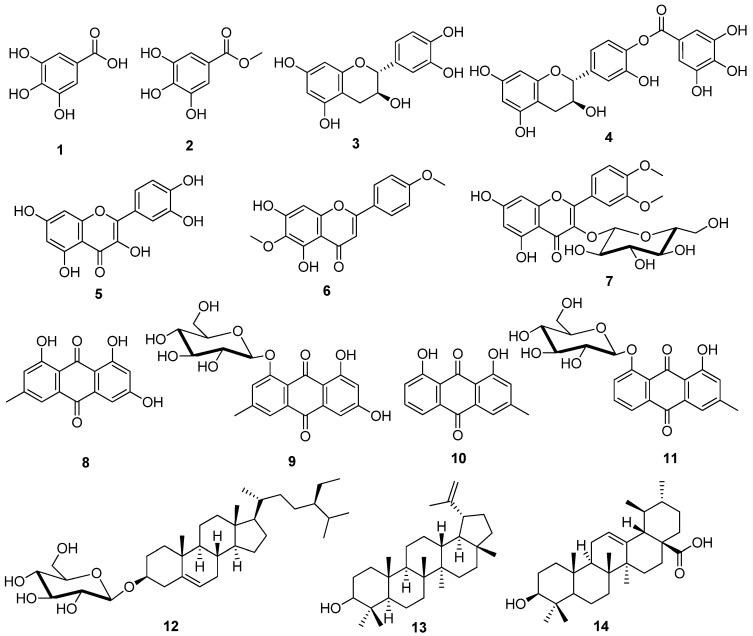
Chemical structures of previously isolated compounds from *P. austroarabica*; (**1**) gallic acid, (**2**) methyl gallate, (**3**) catechin, (**4**) catechin-4′-*O*-gallate, (**5**) quercetin, (**6**) pectolinarigenin, (**7**) dillenetin-3-*O*-glucoside, (**8**) emodin, (**9**) emodin-8-*O*-glucoside, (**10**) chrysophanic acid, (**11**) chrysophanic acid-8-*O*-glucoside, (**12**) *β*-sitosterol-3-*O*-glucoside, (**13**) lupeol, (**14**) ursolic acid.

Cancer is considered a major cause of death and a serious hindrance affecting life spans around the world. The remarkable causes of cancer deaths in males are liver, lung, and gastric cancer. On the other hand, breast, lung, and colorectal cancers are the principal causes among females [1,2]. In 2020, global estimations of new cancer cases and cancer deaths were 19.3 and 10 million, respectively [3,4,5]. The tendency of controlling cancer includes surgery, radiation therapy, or chemotherapy [6]. Natural products present a noteworthy chemical diversity and are considered as a golden mine regarding bioactive metabolites [7]. Between 1981 and 2019, about 25% of the newly approved anti-cancer drugs were naturally-derived [8,9]. The diversity of natural products is the most important factor that inspires the discovery of new anticancer drugs [10]. Among the widely used anticancer therapies are vincristine, etoposide, irinotecan, and paclitaxel. Obviously, camptothecin and taxol are considered the most felicitous examples [11,12,13]. Until now, their succession has endured as a series of new camptothecin derivatives with better properties [14,15]. Loranthaceae is one of the largest families that comprise about 70 genera and 1000 species. The family of Loranthaceae is largely distributed in a pantropical zone in the Southern Hemisphere and is widely used in complementary and alternative cancer therapy. Among the mistletoes belonging to the family of Loranthaceae is *Phragmanthera austroarabica.* It is broadly distributed in Arabian Peninsula, especially in Saudi Arabia and Yemen [16]. *Phragmanthera austroarabica* is a semi-parasitic plant. It is a perennial green mistletoe that directly binds itself to another plant through a haustorium. A haustorium is a very small modified root which composes a morphological and physiological connection between the parasite and the host [17]. Accordingly, the structure of the plant is mainly made of a stem and leaves. It is characterized by isobilateral mesophyll where the supporting collenchyma occurs below the vascular bundle. Stem axial parenchyma is of two types: paratracheal and diffused apotracheal [18]. For many years, *P. austroarabica* had been used in many regions of Africa and Asia to treat microbial and viral infections [19]. *P. austroarabica* is traditionally used for controlling hyperglycemia in Saudi Arabia [20]. There are no sufficient studies investigating the chemical composition and biological activities of *P. austroarabica.* From data reported in literature, it was found that *n*-butanol fraction of *P. austroarabica* showed a potent cytotoxic activity against cervical HeLa cell line while the methanolic extract exhibited significant cytotoxic activity against liver HepG2 and breast MCF-7 cell lines [21]. The methanolic crude extract of *P. austroarabica* ameliorated seizures via the enhancement of neurons’ survival, suppression of necrotic pyknosis, the elevation of glutathione levels, and reduction in malondialdehyde levels in pentylenetetrazole-induced kindling in mice [22]. Our previous studies involved the isolation and identification of a number of anti-inflammatory and antioxidant compounds from *P. austroarabica* which are illustrated in Figure 1 [22,23,24,25].

Gallic acid, methyl gallate, and catechin are among the significant constituents of the family of Loranthaceae in general and are the major bioactive metabolites existing in *P. austroarabica* in particular. They are characterized by cytotoxic, antioxidant, and anti-inflammatory activities [24,25,26,27,28]. Many studies declared that there is a clear correlation between antioxidant and cytotoxic activity against proliferative cells [29,30,31,32]. Additionally, previous reports on natural products declared the possible correlation between anti-inflammatory potential and cytotoxic effect [33,34,35,36]. In parallel, methyl gallate was reported to be a potent antioxidant that inhibits oxidative stress in human adipocytes [35], attenuates doxorubicin-induced cardiotoxicity in rats [35], and suppresses the growth of different types of cancer [36,37]. Methyl gallate and gallic acid exhibit anti-inflammatory properties by blocking the activation of NF-κB [37,38,39,40]. Gallic acid has been reported to prevent the development and progression of various types of cancers inducing apoptosis through Janus kinase 2/signal transducer and activator of transcription 3 (JAK2/STAT3) signaling pathways [41,42] while catechin induced caspase-mediated apoptosis targeting phosphatidylinositol-3-kinase and the protein kinase B (PI3K/AKT) downstream pathway [43]. Catechins are strong anti-inflammatory agents that play a crucial role in the improvement of neurodegenerative and liver disease, metabolic, lung, and GIT disorders [44].

To date, there is no information about the effect of *P. austroarabica* extract on an animal model of breast cancer. Additionally, no previous studies focused on the quantification of its major bioactive metabolites. Accordingly, in the present work, we assessed the cytotoxic effect of the methanolic extract of *P. austroarabica* as well as the pure compounds gallic acid, methyl gallate, and catechin, against ovarian (A2780), prostate (PC-3), breast (MDA-MB-231), and lung (A549) cancer cell lines. This study was also oriented to examine the possible effect of the extract on the tumor mass, apoptotic and proapoptotic gene markers, hematological parameters, and histopathological examination in solid Ehrlich carcinoma (SEC) mice. Moreover, HPTLC quantification of the major bioactive metabolites, gallic acid, catechin, and methyl gallate was a topic of interest.

## 2. Materials and Methods

### 2.1. Plant Material and Extraction Process

*Phragmanthera austroarabica* was previously collected in 2019 from South Abha, in the Southwestern part of Saudi Arabia. The plant was identified by Dr. Nahed M. Wally, Faculty of Science, King Abdulaziz University, and voucher samples were kept and given the code No. 2019-PU. For the extraction process, 50 g fresh weight of *P. austroarabica* was air-dried, finely ground, and then soaked with methanol (3 × 1000 mL) at 25 °C. The total methanolic extract was concentrated under a vacuum to obtain 3.27 g of crude extract of *P. austroarabica*.

### 2.2. Determination of Gallic Acid, Catechin, and Methyl Gallate in Methanolic Extract of P. austroarabica Using HPTLC Analysis

#### 2.2.1. Preparation of Standard Solutions of Gallic Acid, Catechin, and Methyl Gallate

Analytical standards of methyl gallate, catechin, and gallic acid (≥98%; Merck™, Darmstadt, Germany) were used. An amount of 10 mg of each standard was used to prepare a methanolic mixture of standard solution at a concentration of 1 mg/mL. Then, the mixed standard solution was kept in a refrigerator till the construction of the calibration curve.

#### 2.2.2. Analysis Conditions and Construction of Calibration Curves

According to the requirements of the International Council on Harmonization (ICH) guidelines [36], different concentrations of the methanolic mixture of gallic acid, catechin, and methyl gallate were applied using A CAMAG^®^ (Muttenz, Switzerland) Linomat V controlled with CAMAG winCats™ software version 1.4.4. (CAMAG, Muttenz, Switzerland). A plate of silica gel 60 F_254_ (Merck™, Darmstadt, Germany) with the dimensions of 20 cm × 10 cm was used. The conditions were adjusted as follows: slit dimension of 6 mm in length and 0.1 mm in width, a distance between tracks of 10.5 mm, the distances from both x-axis and y-axis were 10 mm, and the rate of application used was 15 μL/second. The methanolic solutions were applied in triplicate. The plates were developed in a twin-trough chamber saturated with a solvent mixture consisting of chloroform–methanol–water (3:7:0.3) containing 1 % (*v*/*v*) glacial acetic acid. Development was performed after saturation for 20 min. After 15 min, the plates were air-dried, and then R_f_ was calculated. The spots were effectively separated with a suitable R_f_ at 0.53, 0.65, and 0.87 for gallic acid, catechin, and methyl gallate, respectively. Quantification was assessed using a CAMAG TLC Scanner III densitometer and CATS version 4 X software in the absorption mode using a deuterium source. Quantification was performed at different wavelengths of λ = 254, 280, and 366 nm. The wavelength corresponding to the best validation parameters was used for the construction of the calibration curve

#### 2.2.3. Plant Sample Assay

For the determination of gallic acid, catechin, and methyl gallate concentration in the methanolic crude extract of *P. austroarabica*, 100 mg of the dry extract was dissolved, and transferred to a 100 mL volumetric flask. Methanol was added to complete the total volume. Different solutions of both sample and the methanolic mixture of standards were applied under the previously described conditions. Finally, different band areas were recorded.

### 2.3. Assessment of In Vitro Cytotoxic Activity of P. austroarabica

#### 2.3.1. MTT Assay

Ovarian “A2780”, prostate “PC-3”, breast “MDA-MB-231”, and lung “A549” cancer cell lines were collected from the “National Cancer Institute in Cairo and grown in RPMI-1640 medium L-Glutamine”. The cells were grown in “10% fetal bovine serum (FBS) and 1% penicillin-streptomycin”. On the second day, cells were cultured in triplicates on a 96-well plate at a density of 5 × 10^4^ cells. This was followed by incubation with the crude extract of *P. austroarabica* and samples of pure compounds catechin, gallic acid, and methyl gallate at 0.1, 1, 10, 50, and 100 µg/mL, and cell viability was determined using MTT solution. An ELISA microplate reader (BIO-RAD microplate reader, model iMark, Japan) was used to measure the absorbance. Cell viability was measured by comparing the absorbance at each well to the control group, and the IC_50_ values were recorded [45].

#### 2.3.2. Annexin V/PI Staining and Cell Cycle Flow Cytometry

MDA-MB-231 cells were incubated overnight in 6-well culture plates (3–5 × 10^5^ cells/well) and then treated with crude extract of *P. austroarabica* (IC_50_ = 19.8 µg/mL, 48 h). After that, the cells were incubated in a 100 µL solution of Annexin binding buffer “25 mM CaCl_2_, 1.4 M NaCl, and 0.1 M Hepes/NaOH, pH 7.4” in the dark for 30 min with “Annexin V-FITC solution (1:100) and propidium iodide (PI) at a concentration equivalent to 10 g/mL.” The labelled cells were then extracted using the Cytoflex FACS machine (Beckman Coulter Inc., Brea, CA, USA). Data were analyzed using CytExpert software [46,47].

#### 2.3.3. Gene Expression Analysis Using RT-PCR

To investigate the apoptosis-inducing activity in MDA-MB-231 cells, gene expression levels of P53, Bax, PUMA, Caspapses-3,8,9, and Bcl-2 were assessed. MDA-MB-231 cells were treated with crude extract of *P. austroarabica* (IC_50_ = 19.8 µg/mL, 48 h). Then, an RT-PCR reaction was performed following routine work, and the results were given in cycle thresholds (Ct) and ΔΔ Ct for calculating the relative quantities of each gene, as previously described [48].

#### 2.3.4. Assessment of Caspase 3/7 Activity

Caspase 3/7 activity in untreated and treated MDA-MB-231 cells (with *P. austroarabica;* IC_50_ = 19.8 µg/mL, 48 h) were examined using the cell event Caspasepase-3/7 fluorescence method kit (No.10009135, Molecular probes, Eugene, OR, USA) following the detailed procedure in [49].

#### 2.3.5. Autophagy Evaluation Using Acridine Orange Quantitative Assessment

Autophagic cell death in MDA-MB-231 cells treated with *P. austroarabica* (IC_50_ = 19.8 µg/mL, 48 h) was quantitatively assessed using acridine orange lysosomal stain coupled with flowcytometric analysis following the previously mentioned procedure [50].

### 2.4. In Vivo Study

#### 2.4.1. Animals

Forty male Swiss albino mice (body weight range: 21–28 g) were used. The mice were kept in a clean and hygienic environment with a normal day/night cycle. Prior to the experiment, mice were subjected to a ten-day period of adaptation to the study conditions. “The experimental protocol was permitted by the Research Ethics Committee (Approval number 202109RA2), Faculty of Pharmacy, Suez Canal University”.

#### 2.4.2. Experiment Design

Mice were equally and randomly divided into four groups: “normal control, SEC control, SEC+ *P. austroarabica*, SEC+5-FU”. SEC cells (1 × 10^6^ tumor cells/mouse) were implanted subcutaneously into the right thigh of the hind limb, and tumor masses were beginning to appear after ten days of tumor cell inoculation. During the experiment duration, seven doses (50 mg/Kg BW, IP) of the *P. austroarabica* and 5-FU (4.2 mg/Kg BW, IP) were used [46,51]. The weight and volume of the solid tumor masses were measured by a digital Vernier clipper (Tricle Brand, Shanghai, China) using the equation; V = (L × W × W)/2, where L is the length and W is the width of tumor mass. At the end of the procedure, animals of different groups were sacrificed and blood samples were collected. CBC parameters, liver enzymes ALT, AST, and kidney parameters of urea and creatinine were measured. Kidney and liver tissues were stained with Hematoxylin and Eosin. A light microscope was used for the histopathological examinations.

## 3. Results and Discussion

### 3.1. Simultaneous Determination of Gallic Acid, Catechin, and Methyl Gallate in a Methanolic Crude Extract of P. austroarabica Using High-Performance Thin Layer Chromatography (HPTLC)

**Figure 2:**
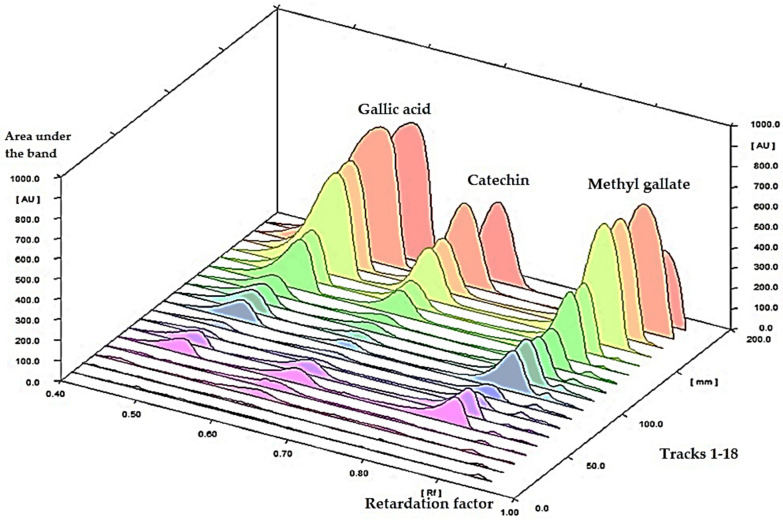
HPTLC spectrum of a standard mixture of gallic acid, catechin, and methyl gallate scanned at λ = 280 nm.

The HPTLC quantification of the major bioactive metabolites, gallic acid, catechin, and methyl gallate, was performed. The spectra of different concentrations of a standard mixture of gallic acid, catechin, and methyl gallate are represented in Figure 2. Upon scanning the chromatographic plate at multi-wavelengths, it was found that λ = 280 nm is corresponding to the highest sensitivity; accordingly, the assessment method and validation parameters (e.g., linearity, precision, accuracy, stability, and limits of quantification and detections) were assessed at 280 nm.

#### 3.1.1. Linearity

A linear relationship for each standard was detected over the concentration range recorded in Table 1. The correlation coefficients (R^2^) were 0.99, and the linear regression equations for gallic acid, catechin, and methyl gallate are expressed in Table 1.

#### 3.1.2. System Precision

The system precision was verified by the determination of the band area corresponding to a standard mixture at a concentration of 1 mg/mL, applied in triplicate. The value of percent-relative standard deviation (%RSD) for each standard was calculated (Table 1).

#### 3.1.3. Method Precision

The method precision was checked by the injection of the plant extract under the same procedure as described above. Five measurements were performed. The low value of % relative standard deviation (RSD) revealed the method’s precision (Table 1).

#### 3.1.4. Accuracy

The accuracy was assessed under the same conditions by scanning a fortified sample; the known sample was combined with a certain concentration of a standard mixture of gallic acid, catechin, and methyl gallate solution (Table 1).

#### 3.1.5. Limits of Detection and Quantification

The limit of detection was computed through the equation of 3 σ/S while the limit of quantification was assessed through the equation of 10 σ/S. The σ refers to the standard deviation of the response and S refers to the slope of the calibration curve (Table 1).

#### 3.1.6. Analytical Solution Stability

The stability of a standard mixture was assured by comparing the experimental results performed with those at ambient temperature for 2 days and after storage of them at 4 °C for 10 days.

#### 3.1.7. Sample Analysis

**Figure 3:**
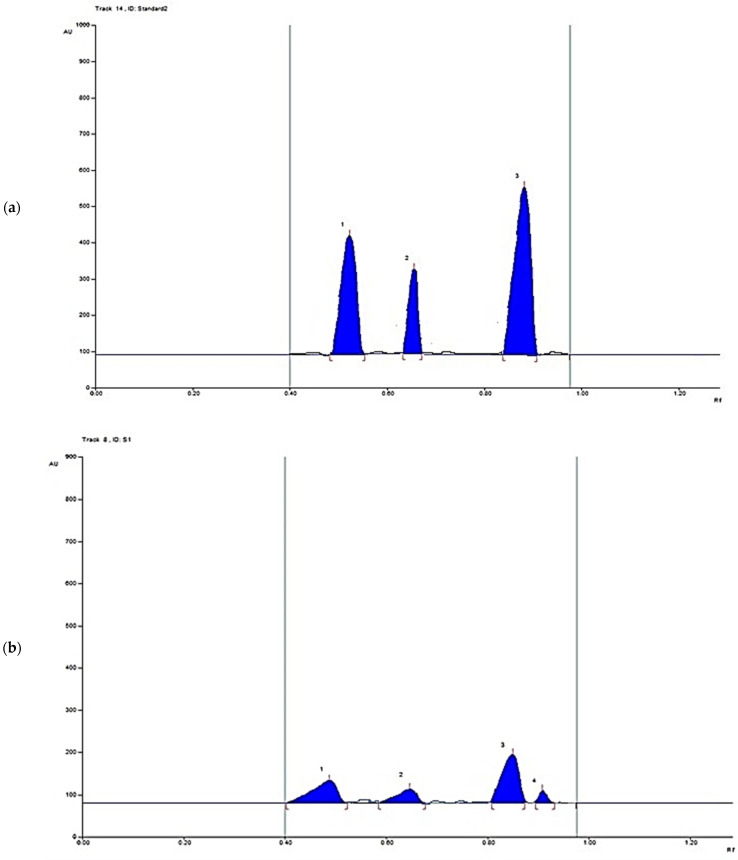
(**a**) HPTLC chromatogram of 1 µg/band of gallic acid, catechin, and methyl gallate scanned at λ = 280 nm; (**b**) HPTLC chromatogram of 10 µg per band of *P. austroarabica* extract scanned at λ = 280 nm.

The suggested method was utilized for the determination of the gallic acid, catechin, and methyl gallate in *P. austroarabica* which was applied to bands in triplicate. The bands were scanned at λ 280 nm (Figure 3). The concentrations were found to be 14.5, 6.5, and 43.6 mg/g of plant dry extract for methyl gallate, catechin, and gallic acid, respectively.

### 3.2. In Vitro Activities P. austroarabica Extract

#### 3.2.1. Cytotoxicity of *P. austroarabica* against PC-3, MDA-MB-231, A2780, and A549 Cancer Cell Lines Using MTT Assay

The crude extract of *P. austroarabica,* as well as the pure compounds of gallic acid, methyl gallate, and catechin, were screened for their cytotoxicity against a panel of cancer cell lines, such as prostate “PC-3”, breast “MDA-MB-231”, ovarian “A2780”, and lung “A549” cell lines using MTT assay. According to the US NCI (National Cancer Institute) plant screening program guidelines, a crude extract is considered to have in vitro cytotoxic activity if the IC_50_ value after incubation between 48 and 72 h is less than 20 µg/mL. While the pure compounds have in vitro cytotoxic activity if the IC_50_ value is less than 4 µg/mL [52,53]. Cytotoxicity results, as seen in Table 2, showed that *P. austroarabica* extract exhibited promising cytotoxicity against MDA-MB-231 cells with IC_50_ value of 19.8 μg/mL compared to the tested samples of pure compounds with IC_50_ values range (15.36–35.7 μg/mL). Additionally, *P. austroarabica* extract was non-toxic against normal breast cells MCF-10A with IC_50_ values of 47.26 μg/mL. These results agreed with the reported antioxidant activity of *P. austroarabica* extract, and this may elucidate the cytotoxic activity against cancer cells [24]. Additionally, the more prominent effects exhibited by *P. austroarabica* extract could be attributed to the combined activity of its major and other chemical constituents. The plant extract accumulates a number of active metabolites that were previously reported to possess antiproliferative effects against breast cancer cells. For example, *β*-sitosterol 3-*O*-glucoside was reported to suppress tumor growth through upregulating miR-10a expression as well as deactivating the PI3K-Akt-signaling pathway. Accordingly, it was recommended as a significant breast anticancer agent [54]. Lupeol inhibited the invasion of MDA-MB-231 cells through the suppression of the protein expression of COX-2, MMP-2, and MMP-9 [55]. Ursolic acid a showed proliferative inhibitory effect through downregulating Nrf2 via the Keap1/Nrf2 pathway and EGFR/Nrf2 pathway in MDA-MB-231 cells [56]. Both the anthraquinones emodin and chrysophanic acid were reported to inhibit invasion and metastasis of human breast cancer MDA-MB-231 cells [57,58]. The well-known flavonoid quercetin was reported to induce apoptosis in breast cancer cells by the suppression of Twist via the p38MAPK pathway [59], and in another study, it revealed inhibition of calcium-dependent urokinase activity and, hence, proved to be an effective antimetastatic agent [60]. Finally, catechin 4- gallate, when combined with 4-hydroxytamoxifen, disclosed synergistic cytotoxicity in MDA-MB-231 cells [61,62].

#### 3.2.2. *P. austroarabica* Treatment Induced Apoptosis in MDA-MB-231 Cells

**Figure 4:**
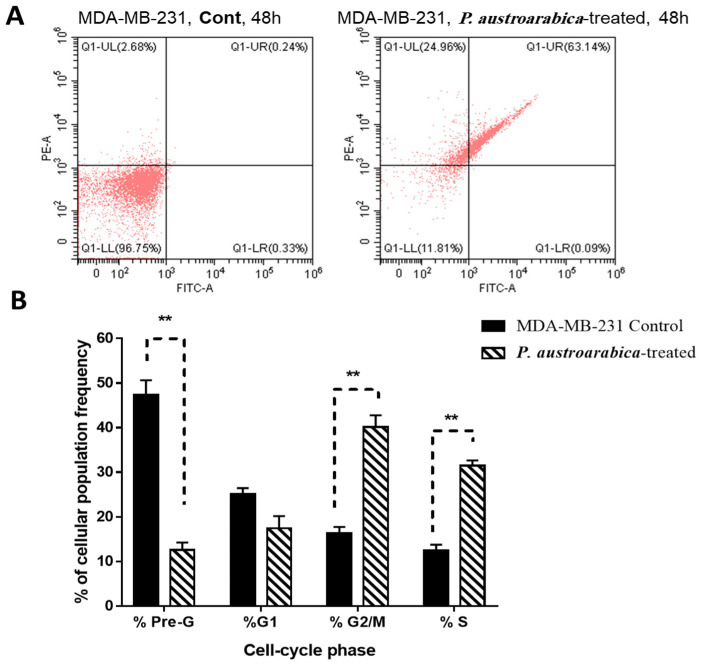
Flow cytometry analysis of MDA-MB-231 untreated and treated cells with *P. austroarabica* crude extract (IC_50_ = 19.8 µg/mL, 48 h). (**A**) Annexin V/PI staining for apoptosis/Necrosis assessment; (**B**) DNA content-cell cycle analysis. “Values are expressed as Mean ± SD of three independent values. ** (*p* ≤ 0.001) significantly different between treated and control using unpaired t-test in GraphPad prism”.

Cytotoxic activity of *P. austroarabica* against MDA-MB-231 cells was investigated for its mechanism for apoptosis-induction using Annexin V/PI staining. As seen in Figure 4a, the extract induced total apoptotic cell death by 63.23% compared to 0.57% in the untreated control cells. Additionally, it induced necrotic cell death by 24.96% compared to 2.68%, these findings showed that the *P. austroarabica* treatment induced cell death by apoptosis by 111-fold change and necrosis by 9.31-fold change. DNA content-aided cell cycle analysis was carried out to determine the cell population at each phase. This step aimed to determine the cell cycle at which the cell proliferation was arrested. As seen in Figure 4b, *P. austroarabica* treatment significantly increased the cell population at G2/M and S-phases by 2.46-fold and 2.5-fold change compared to untreated control, so its treatment significantly induced the cell cycle arrest at G2/M and S-phases in MDA-MB-231 cells.

#### 3.2.3. *P. austroarabica* Treatment Affected Gene Expression Analysis of Apoptosis-Related Genes

To further validate the apoptosis induction of *P. austroarabica* treatment in MDA-MB-231 cells, RT-PCR for the apoptosis-related genes was performed in the untreated and treated cells. As seen in Table 3, *P. austroarabica* treatment upregulated the P53 gene by 9.28-fold, the PUMA gene by 9.39-fold, the Bax gene by 7.39-fold, and caspases 3, 8, 9 by 10.36, 20.8, 12.39-fold, respectively. In contrast, it downregulated the Bcl-2 gene by 0.53-fold. This behavior of apoptosis-induction in MDA-MB-231 cells upon treatment agreed with the routine results [63,64] of proving apoptosis induction.

#### 3.2.4. *P. austroarabica* Treatment Activated Caspase 3/7 Activity

**Figure 5:**
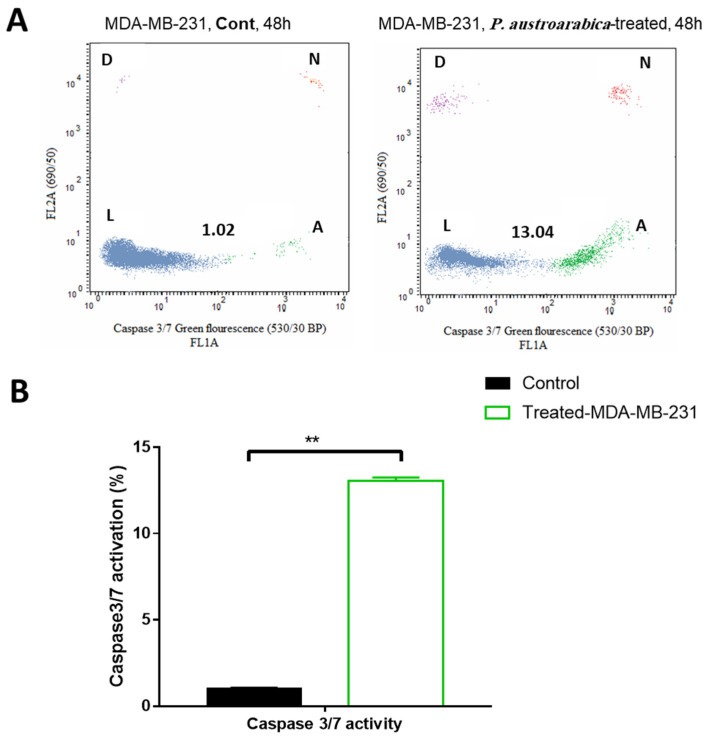
(**A**) Caspase 3/7 inhibitory activity in cancer MDA-MB-231 cells was treated with *P. austroarabica* crude extract (IC_50_ = 19.8 µg/mL, 48 h) using the “CellEvent^®^ Caspase-3/7 Green Flow Cytometry kit”, where “L, viable cells; A, apoptotic cells; N, necrotic cells; and D, dead cells”; (**B**) Bar presentation for comparison of apoptotic cancer cells due to active caspases 3/7 of the tested extract. The data are expressed as the mean ± SEM of three independent experiments in triplicate. ** (*p* ≤ 0.001) significantly different between treated and control using unpaired t-test in GraphPad prism”.

Apoptosis is a form of cell death programmed by the family of cysteine protease. In response to the various cell death stimuli, a large irreversible proteolytic cascade is triggered and subsequently generated. The effector caspases activity has been evaluated to determine the apoptotic pathway initiated by the tested compounds, using the “Cell Event™ Caspase-3/7 Green Detection kit”. As seen in Figure 5, the *P. austroarabica* treatment induced a higher percentage of MDA-MB-231 cancer cell death via apoptosis with a percentage of 13.04% than the untreated cells at 1.02%. Hence, apoptotic cell death was triggered by activation of effectors 3 and 7 caspases in mDA-MB-231 cells

#### 3.2.5. *P. austroarabica* Induced MDA-MB-231 Cell Death through Autophagy

**Figure 6:**
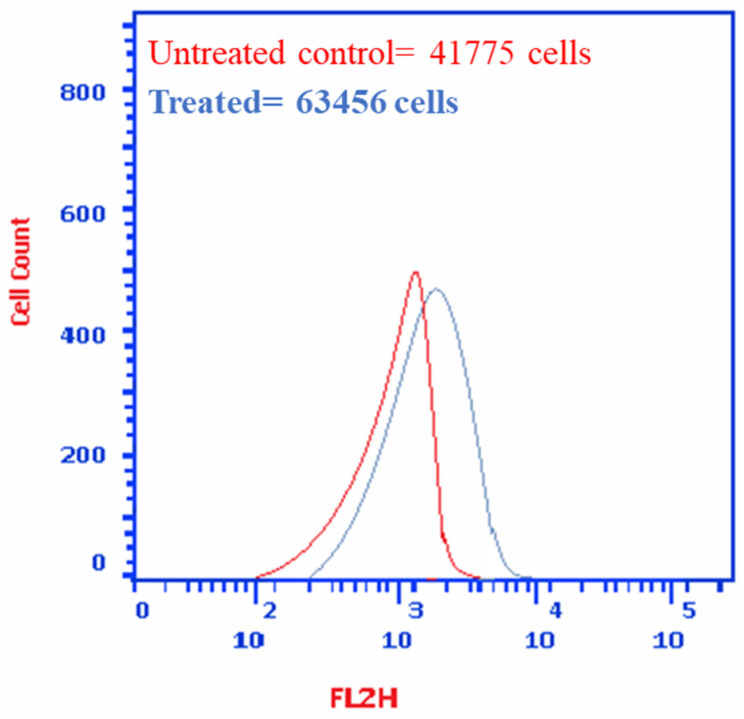
Cell death through autophagy in untreated and treated MDA-MB-231 cells with *P. austroarabica* crude extract (IC_50_ = 19.8 µg/mL, 48 h) using the acridine orange lysosomal stain coupled with the flow cytometric analysis. Red: Negative control (untreated), Blue: treated cells.

As seen in Figure 6, cell death in MDA-MB-231 cells through autophagy was assessed using acridine orange lysosomal stain. *P. austroarabica* induced autophagy by 1.5-fold, the cell population in the treated group was 63,456 cells compared to 41,775 cells in the untreated control.

### 3.3. In Vivo Study of P. austroarabica against Solid Ehrlich Carcinoma

Solid weight masses and volumes of solid Ehrlich carcinoma (SEC) were measured at the end of the experiment. As seen in Table 4, an increase in solid tumor weight of 203 mg was observed via tumor proliferation. Upon treatment with *P. austroarabica* extract and 5-FU, a significant reduction in the solid tumor mass of 96.8 mg and 78.3 mg, respectively, was noticed. Accordingly, treatments with *P. austroarabica* extract and Fluorouracil (5-FU) significantly inhibited tumor proliferation by 54% and 64%, respectively, by reducing the tumor volume from 357 mm^3^ in untreated control to 169 mm^3^ and 126 mm^3^.

In SEC-bearing mice, hematological and biochemical parameters are summarized in Table 4. In the SEC control, all CBC parameters were altered, with Hb content and RBCs significantly reduced to 3.69 (g/dL) and 2.19 (10^6^/L), respectively. While the WBC count elevated to 6.63 (10^3^/L) compared to the normal control. Reduced hemoglobin and RBC levels, as well as an increase in WBC counts, are common side effects of tumor progression [49,65]. Treatment with *P. austroarabica* extract almost restored CBC levels to normal. It increased Hb (7.12 g/dL), RBC (5.01 × 10^6^/L), and WBC (4.01 × 10^3^/L) levels. Interestingly, our results support those of a previous study [51,66] which illustrated the ameliorative effect in the hematological parameters after treatment with the tested compound.

**Figure 7:**
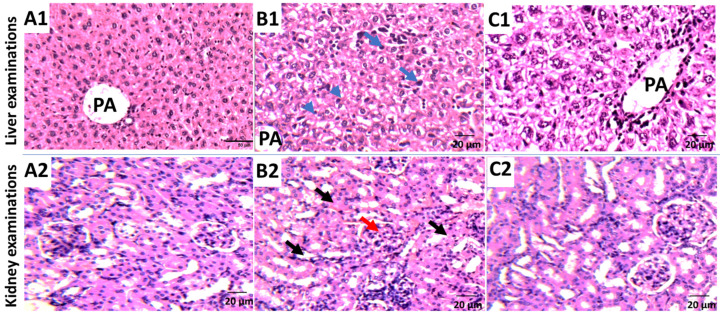
Histopathological examinations of liver and kidney tissues in (**A1**,**A2**) Normal control; (**B1**,**B2**) SEC control; (**C1**,**C2**) *P. austroarabica*-treated SEC group. Portal area (PA); chronic inflammation (blue arrow); hydropic degeneration (Arrow heads). Normal glomeruli (Black arrows), urinary spaces, and tubules (Red arrows).

In terms of biochemical parameters, liver enzymes (ALT and AST) were significantly elevated to 66.51 and 92.5 (U/L), respectively, when compared to normal mice (43.6 and 46.5 U/L), respectively. Treatment with *P. austroarabica* substantially normalized liver enzymes to be 52.4 and 56.1 U/L, respectively, indicating a significant inhibition of hepatocellular toxicity caused by tumor proliferation. Additionally, kidney parameters, urea, and creatinine levels were deteriorated in cancer groups, while *P. austroarabica* treatment retained their levels to be 30.3 and 0.87 (mg/dL), respectively, nearly at normal control. In agreement with hematological and biochemical examinations, as seen in Figure 7, histopathological examinations of liver and kidney tissues exhibited improvement in their structure to be near normal with less hydropic degeneration and inflammation [66]. Taken together with improvement in the tumor volume and weight, treatment of SEC mice with *P. austroarabica* extract improved both hematological and biochemical parameters.

## 4. Conclusions

In conclusion, the present study handled the HPTLC quantification of gallic acid, catechin, and methyl gallate. It is a platform for cytotoxic activity of *P. austroarabica* extract against MDA-MB-231 cells with IC_50_ value of 19.8 μg/mL compared to the tested samples of pure compounds with potent cytotoxic activities with IC_50_ values range (15.36–35.7 μg/mL). For apoptosis-investigation, *P. austroarabica* treatment induced cell death by apoptosis by 111-fold change and necrosis by 9.31-fold change, and it activated the proapoptotic genes markers, while it inhibited the antiapoptotic gene. Moreover, in vivo results exhibited inhibition in the tumor volume and weight, and the treatment of SEC mice with *P. austroarabica* extract improved both hematological and biochemical parameters with amelioration in the liver and kidney histopathology being near normal. Taken together, *P. austroarabica* extract exhibited promising anti-cancer activity through apoptosis-induction. Hence, further future work of the formulation of crude extract and purified compounds will be handled and correlated with their biological activity. In addition, semisynthetic derivatives of methyl gallate, catechin, and gallic acid will be developed and assessed as target-oriented chemotherapeutic agents against breast cancer.

## Figures and Tables

**Table 1:** Validation parameters of the method for the estimation of methyl gallate, catechin, and gallic acid using HPTLC with densitometer scanning.

Validation Parameters	Scanned at λ 280 nm		
	Methyl Gallate	Catechin	Gallic Acid
Linearity range (µg/band)	0.8–9	0.4–9	0.8–9
Correlation coefficient (R^2^)	0.99	0.99	0.99
Regression equation	Y = 5926.9X + 1684.1	Y = 2175.9X + 1352.9	Y = 6831.9X − 1984.2
Limit of detection (µg/band)	0.1	0.09	0.11
Limit of quantification	0.31	0.29	0.34
System precision [%RSD]	3.72	3.27	2.74
Method precision [%RSD]	3.29	1.97	3.91
% Recovery	95.89	95.58	96.64
Conc. (mg/g extract)	14.5	6.5	43.6

Y: band area; X: concentrations of gallic acid, catechin, or methyl gallate; RSD: relative standard deviation.

**Table 2:** Cytotoxic activity of crude extract of *P. austroarabica*, gallic acid, methyl gallate, and catechin against panel of cancer cell lines using MTT assay.

Sample	IC_50_ * (μg/mL)				
	PC-3	MDA-MB-231	A2780	A549	Normal MCF-10A
*P. austroarabica*	36.9 ± 1.89	19.8 ± 0.76	38.6 ± 2.03	NA	47.26 ± 2.03
Gallic acid	22.15 ± 1.39	29.6 ± 0.96	33.41 ± 1.21	17.82 ± 0.92	≥50
Methyl gallate	15.36 ± 1.02	21.98 ± 0.95	35.7 ± 1.97	19.6 ± 2.12	≥50
Catechin	19.21 ± 0.78	21.26 ± 1.31	31.8 ± 2.19	26.39 ± 2.01	≥50
5-FU	2.64 ± 0.35	1.59 ± 0.36	3.64 ± 0.45	4.26 ± 0.64	49.65 ± 2.04

* IC_50_ are expressed as mean ± SD of three independent trials and were calculated using GraphPad prism.

**Table 3:** Fold of change of apoptosis-related genes in untreated control and treated MDA-MB-231 with *P. austroarabica* crude extract (IC_50_ = 19.8 µg/mL, 48 h).

Sample	Gene Expression (Fold Change) *						
	Pro-Apoptotic Genes						Anti-Apoptotic Gene
	P53	PUMA	Bax	Casp-3	Casp-8	Casp-9	Bcl-2
Cont./ MDA-MB-231	1						
*P. austroarabica* MDA-MB-231	9.28 ± 1.38	9.39 ± 1.76	7.39 ± 1.38	10.36 ± 1.98	2.08 ± 0.98	12.39 ± 1.67	0.53 ± 0.01

* Fold of change is calculated by 2^—ΔΔCT^, where ΔΔCT is the difference between mean values of genes CT values in the treated and control groups.

**Table 4:** Hematological and biochemical parameters in the normal, untreated, and treated SEC-bearing mice.

Parameters		Normal Control	SEC Control	SEC + *P. austroarabica*	SEC + 5-FU
Anti-tumor potentiality	Tumor weight (mg)	--	203.6 ± 4.26	96.8 ± 2.34	78.3 ± 2.38
	Tumor volume (mm^3^)	--	356.9 ± 22.3	168.8 ± 19.8	126.2 ± 18.6
	Tumor inhibition ratio (TIR%)	--	--	54.26 ± 1.36	64.7 ± 1.65
Hematological parameters	Hb (g/dL)	8.16 ± 0.67	3.69 * ± 0.6	7.12 ^#^ ± 0.64	7.89 ^#^ ± 0.54
	RBC’s count (×10^6^/μL)	5.98 ± 0.56	2.19 * ± 0.54	5.01 ^#^ ± 0.56	5.21 ^#^ ± 0.44
	WBC’s count (×10^3^/μL)	3.27 ± 0.34	6.63 * ± 0.41	4.01 ^#^ ± 0.55	3.69 ^#^ ± 0.69
Liver and kidney parameters	ALT (I/U)	43.3 ± 1.23	66.5 * ± 1.99	52.4 ^#^ ± 1.4	48.5 ^#^ ± 1.7
	AST (I/U)	46.5 ± 0.78	92.6 * ± 1.45	56.1 ^#^ ± 2.0	50.8 ^#^ ± 1.5
	Urea (mg/dL)	23.2 ± 1.06	41.3 * ± 1.01	30.3 ^#^ ± 1.36	30.3 ^#^ ± 1.01
	Creatinine (mg/dL)	0.76 ± 0.02	1.01 ± 0.17	0.87 ± 0.01	0.64 ± 0.06

Mean ± SEM values of mice in each group (n = 6). * Values are significantly different (*p* ≤ 0.05) between SEC control and normal group, while ^#^ values are significantly different (*p* ≤ 0.05) between Treated SCE and untreated SEC mice using un-paired test in GraphPad prism.

## Data Availability

The data presented in this study are available in the article.
